# Performance Evaluation of Highly Admixed Tanzanian Smallholder Dairy Cattle Using SNP Derived Kinship Matrix

**DOI:** 10.3389/fgene.2019.00375

**Published:** 2019-04-26

**Authors:** Fidalis D. N. Mujibi, James Rao, Morris Agaba, Devotha Nyambo, Evans K. Cheruiyot, Absolomon Kihara, Yi Zhang, Raphael Mrode

**Affiliations:** ^1^USOMI Limited, Nairobi, Kenya; ^2^Nelson Mandela African Institution of Science and Technology, Arusha, Tanzania; ^3^International Livestock Research Institute, Nairobi, Kenya; ^4^Department of Animal Production, College of Agriculture and Veterinary Sciences, University of Nairobi, Nairobi, Kenya; ^5^Badili Innovations Limited, Nairobi, Kenya; ^6^College of Animal Science and Technology, China Agricultural University, Beijing, China; ^7^Scotland’s Rural College, Edinburgh, United Kingdom

**Keywords:** SNP, dairy, performance, cluster, smallholder, admixture, EBV, BLUP

## Abstract

The main purpose of this study was to understand the type of dairy cattle that can be optimally used by smallholder farmers in various production environments such that they will maximize their yields without increasing the level of inputs. Anecdotal evidence and previous research suggests that the optimal level of taurine inheritance in crossbred animals lies between 50 and 75% when considering total productivity in tropical management clusters. We set out to assess the relationship between breed composition and productivity for various smallholder production systems in Tanzania. We surveyed 654 smallholder dairy households over a 1-year period and grouped them into production clusters. Based on supplementary feeding, milk productivity and sale as well as household wealth status four clusters were described: low-feed–low-output subsistence, medium-feed–low-output subsistence, maize germ intensive semi-commercial and feed intensive commercial management clusters. About 839 crossbred cows were genotyped at approximately 150,000 single nucleotide polymorphism (SNP) loci and their breed composition determined. Percentage dairyness (proportion of genes from international dairy breeds) was estimated through admixture analysis with Holstein, Friesian, Norwegian Red, Jersey, Guernsey, N’Dama, Gir, and Zebu as references. Four breed types were defined as RED–GUE (Norwegian Red/Friesian–Guernsey; Norwegian Red/Friesian–Jersey), RED–HOL (Norwegian Red/Friesian–Holstein), RED–Zebu (Norwegian Red/Friesian–Zebu), Zebu–RED (Zebu–Norwegian Red/Friesian) based on the combination of breeds that make up the top 76% breed composition. A fixed regression model using a genomic kinship matrix was used to analyze milk yield records. The fitted model accounted for year-month-test-date, parity, age, breed type and the production clusters as fixed effects in the model in addition to random effects of animal and permanent environment effect. Results suggested that RED–Zebu breed type with dairyness between 75 and 85% is the most appropriate for a majority of smallholder management clusters. Additionally, for farmers in the feed intensive management group, animals with a Holstein genetic background with at least 75% dairy composition were the best performing. These results indicate that matching breed type to production management group is central to maximizing productivity in smallholder systems. The findings from this study can serve as a basis to inform the development of the dairy sector in Tanzania and beyond.

## Introduction

The use of crossbred animals continues to be the basis for most dairy enterprises in Eastern Africa. However, the indiscriminate crossbreeding practiced in these systems produces highly admixed animals with large variability in productivity (Ojango et al., 2014). Additionally, since the breed composition of the animals is unknown, there is often a mismatch between production environment and animal breed type, which often reduces productivity. This situation cannot sustain the growth and expansion of the local dairy sector in many of the countries in the region. With the increased demand for livestock products and the need to bridge productivity gaps in developing countries, poorly planned crossbreeding of locally adapted breeds with imported exotic breeds have been widely adopted yielding animals with unknown breed composition (Weerasinghe et al., 2013). Suitability of these crosses to various production environments is largely unknown.

Anecdotal evidence and previous research suggests that the optimal level of taurine inheritance in crossbred animals lies between 50 and 75% when considering total productivity in terms of fertility, survival, growth rate and milk yield (Bee et al., 2006). However, the mismatch between genotype and environment as a result of unplanned crossbreeding contributes to depress performance mimicking indigenous cattle production (∼1.6 l/day; Mwacharo and Rege, 2002). Even though it is clear that increasing the exotic percentage of cattle results in more milk, the cumulative benefits relative to farmer socio-economic status, input level and production environment are not clear. This study sought to assess the incremental benefit from use of crossbred cattle, given the two sites with varying market orientations and markedly different improved cattle populations.

The study was undertaken in Tanzania, being an emerging dairy region where significant crossbreeding efforts are taking place. The country has a small population of improved dairy animals, (about 800,000) such that the demand for milk currently outstrips available supply. Most dairies and milk processing facilities are running below capacity. According to FAO data series, the quantity of dairy output (milk and butter) in Tanzania has grown by 4.4% per annum, barely keeping up with the population growth rate of about 4.5% since 1980. This has led to stagnation in per capita milk consumption at 39 kg/year (National Bureau of Statistics [NBS], 2007). The supply scenario points to low productivity with a modest annual growth in milk productivity of 1.1% from 160 kg in 1965 to 239 kg/cow in 2010. In view of the above, the government of Tanzania has embarked on developing a national dairy strategic plan with a view of increasing milk production from the current 1.6 billion liters of milk to 8 billion liters. It is estimated that three million head of improved cattle will be required to achieve this target in 12 years, starting from 2014. This will be a tall order given the modest increases of about 400,000 head of improved cattle between 1984 and 2005 (Swai et al., 1992; Kurwijila and Bennett, 2011). Such massive increase in the herd can only be achieved by increasing crossbreeding, especially through innovative use of estrus synchronization and artificial insemination, followed by improved calf management to enable rapid multiplication and increased survival of the desired cattle. Understanding the implications of breed by environment interactions, as this project seeks to do, will modulate the speed at which the milk production target is achieved. Smallholder farmers are the backbone of the dairy sector in Tanzania. It is generally agreed that a successful dairy operation should utilize improved breed types given the low productivity of local zebu cattle. This desire for increased production drives farmers into crossbreeding, the general sense being that a purebred exotic animal isn’t suitable either for a majority of smallholder farmers. However, there is little information or evidence to support what should be the ideal grade cattle for various smallholder production situations (Msanga, 1994). Because there are no planned programs to aid farmers in this grading up process, the resulting animals constitute a mixture of breeds whose composition is unknown; animals that require much more intensive management are as a consequence managed similarly with animals of low genetic potential, which naturally make do with minimum care. Since not all breed types are well adapted to extant production environments, milk yields continue to be low. Knowledge of breed composition is therefore critical in matching breeds to the production environment as well as predicting genetic effects of heterosis (VanRaden and Sanders, 2003).

Pedigree data has been the main source of information for determining breed composition. However, the availability of dense genome-wide single nucleotide polymorphism (SNP) arrays has enabled accurate establishment of kinship and genetic composition of animals in a herd and in their native environments (*in situ*). The use of genetic markers, and especially SNPs in determining breed composition of cattle has attracted great interest in recent years especially in developing countries which are mostly characterized by lack of or incomplete pedigree records (Rege et al., 2001; Gorbach et al., 2010). Previous studies have demonstrated the utility of SNP markers in providing highly reliable estimates of kinship and relationships between animals (Strucken et al., 2017). Additionally, application of SNP markers in deciphering the breed composition of crossbred animals is increasingly gaining popularity. Knowledge on breed composition will be important for farmers who can then start planned crossbreeding since they will know the level of exotic ‘blood’ in their animals. By identifying the exact breed composition of animals and associating this with individual animal productivity, it is envisaged that appropriate recommendations can be made for farmers and others intending to maximize productivity of their enterprises.

The purpose of this study was to determine the differential performance of various dairy genotypes and grade levels under varying resource bases and management clusters in two regions of Tanzania. The results from this study can serve as a basis to inform the development of the dairy sector in Tanzania and Eastern Africa in general.

## Materials and Methods

### Ethics Statement

This study was performed following the International Livestock Research Institute (ILRI) Institutional Animal Care and Use Committee (IACUC) guidelines, with approval reference number 2014.35. Animals were handled by experienced animal health professionals to minimize discomfort and injury.

### Sampling Site Selection and Inclusion Criteria

Data used in this study was obtained from a baseline survey of smallholder dairy farmers in the Northern and Southern highlands of Tanzania. The project covered two sites namely: Rungwe district in Southern highlands and Lushoto district in the Northern highlands that were selected through a stakeholder engagement process. Within each of these sites, wards were selected based on the dairy cattle density data obtained from the regional government offices. Villages were then randomly selected within each selected ward (12 wards in Lushoto and 16 wards in Rungwe). From each of the villages, households were purposively recruited depending on whether they met certain inclusion criteria.

### Inclusion Criteria and Sample Size

To qualify for inclusion in the study, target dairy farmers had to be smallholders rearing between 2 and 10 dairy cows. Qualifying households had to have at least two cows, one of which had to be lactating having calved recently. Additionally, based on farmer knowledge, unrelated animals were recruited to maximize observable breed diversity within the household. Additional criteria for target animals required selected cows to be either pregnant heifers, cows in the third trimester of pregnancy or be a cow that had calved within 3 months of the recruitment date. This increased the chances that recruited cows would be in milk within a significant portion of the study period to allow collection of data on milk yield, calving and reproductive performance. This selection process yielded 654 households which were interviewed by way of a baseline survey regarding general farm and household socioeconomic conditions, animal husbandry and management practices as well as breeding practices among others. In total, 1,255 animals were recruited for the study.

### Production Cluster Characterization

In order to classify and characterize smallholder dairy farmers across the two project sites, we undertook cluster analysis. Farms were grouped based on common characteristics using agglomerative hierarchical clustering. The method groups farms such that individual farms in the same clusters are more alike than they are to farms in other clusters. Cluster analysis was preceded by an exploratory factor analysis (EFA) of all the variables that represented the various themes in the baseline survey. Variables related to livestock feeding and management as well as wealth indicators were considered as relevant variables for inclusion in cluster analysis. We also included variables linked to household endowment with livestock, particularly ownership of lactating cows. Sampling adequacy and data suitability for clustering was measured using the Kaiser–Meyer–Olkin (KMO) statistic. Factor extraction was achieved through principal axis factoring (PAF), to characterize interrelationships between respective variables related to smallholder dairy farming systems. Parallel analysis was used to determine the exact number of factors to be retained. Varimax rotation with Kaiser normalization was used to increase the interpretability of the retained factors. Extracted factors were then subjected to an agglomerative hierarchical clustering procedure using the squared Euclidean distance criterion in conjunction with Ward’s linkage method. The Duda-Hart index and its associated pseudo-T-squared as well as inspection of the clustering dendrogram were used to decide on the optimal number of clusters to retain. Clustering was done using SPSS software (SPSS Inc., Chicago, IL, United States).

### Blood Sampling

Qualified veterinary and animal health personnel undertook blood sampling through jugular venipuncture using approved procedures. Hair samples were collected from the tail switch of the animals. Samples were collected from all animals in the study.

### Genotyping and Quality Control

About 839 animals (490 from Rungwe and 349 from Lushoto) were genotyped using the Geneseek Genomic Profiler (GGP) High Density (HD) SNP array consisting of 150,000 SNPs, while genotypes for the reference breeds were derived from sample sets genotyped using the Illumina HD Bovine Chip (777K SNPs). Since pedigree records were not available for these animals, and in order to aid in breed composition determination, a panel of reference genotypes consisting of Friesian (28 animals), Holstein (63), Norwegian Red (17), Jersey (36), and Guernsey (21), N’Dama (24), East African Zebu (50), and Gir (30) were included in the analysis. A total of 134,295 SNPs were common across study and reference datasets. Data quality control was undertaken using PLINK v 1.9 (Purcell et al., 2007) and included removal of SNPs with less than 90% call rate, less than 5% minor allele frequency (MAF) and samples with more than 10% missing genotypes. A total of 4,324 SNPs were removed, leaving 129,971 SNPs available for analysis. Similarly, eight samples did not meet the above quality thresholds and were removed from the final dataset. The average genotyping rate in the remaining samples was 0.9964. For the purposes of developing a kinship matrix, the SNP data were further validated, excluding SNPs with GC score of less than 60% and those in the sex and mitochondrial chromosomes. Computation of the genomic kinship matrix (**G** matrix) was based on 112,856 SNPs after validation using method one of VanRaden (2008).

### Admixture Analysis and Dairyness

Breed composition of individual animals was estimated using the unsupervised model-based clustering method implemented by the program ADMIXTURE v. 1.3.0 (Alexander et al., 2009). The number of distinct breeds was set to a minimum of 2 and maximum of 9 to reflect the basic cross (indicine and taurine cross) and total number of the populations in the analysis, respectively, given the eight reference breeds. Ten-fold cross-validation (CV = 10) was used, with the error profile subsequently used to determine the most appropriate number of distinct clusters (K), as described by Alexander et al. (2009).

### Daily Milk Yield Data

A total of 539 cows had records on milk yield. About 300 animals either were sold, had dried up or were from farmers who did not collect milk records at all. The data was obtained from individual animals over a period of 7 months. Each animal was visited approximately every 1.5 months for a test day record to be obtained. The analysis of daily milk yield data was undertaken using about 1328 test day records from 539 cows. Test day data ranged between one to six records per animal, with a majority of animals (80%) having less than four records (Table 1). A fixed regression animal model was fitted as shown below (Brown et al., 2016):

**Table 1:** Distribution of the number of test records available for analysis.

Number of	Number	Proportion of
test records	of cows	population
1	118	21.89
2	171	31.73
3	142	26.35
4	99	18.37
5	8	1.48
6	1	0.19

$$y_{\mathrm{tij}}\;=\;{\mathrm{Fixed}}_{\text{i}}\;+\;\sum_{\mathrm{k=0}}^{3}{\text{ϕ}}_{\mathrm{tjkm}}{\text{β}}_{\mathrm{km}}\;+\;{\text{u}}_{\text{j}}\;+\;{\mathrm{pe}}_{\text{j}}\;+\;{\text{e}}_{\mathrm{tij}}$$

where y_tij_ is the test day record of cow j made on day t; Fixed_i_ are the i^th^ fixed effects consisting, year-month of test-day, lactation number (eight levels), and age at calving as a covariate nested within the lactation number, β_km_ are k^th^ fixed regressions coefficients of breed type nested within a herd management group; *u*_j_ and *pe*_j_ are vectors of animal additive genetic and permanent environmental effects, respectively, for animal *j*; ϕ_tjk_ is the vector of the k^th^ Legendre polynomials of order three, for the test day record of cow *j* made on day *t* and e_tij_ is the random residual. The relationship among animals was taken into account in the analysis by fitting a ***G*** matrix, thus the variance of ***u*** was assumed to be equal to var(***u***) = *G*σ^2^_u_. The analysis was carried out using ASREML (Gilmour et al., 2009).

### Breed Type Suitability Assessment

The suitability of breed types for each of the four management clusters characterized was first determined by computing the mean of the raw daily milk yield for each breed type in each management group as well as mean milk production corrected for the fixed effects affecting milk yield fitted in the model. Additionally, the ranking of animals based on their EBVs and breed composition for each management system was also used to determine the best breed type in each management system.

## Results

### Cluster Analysis and Farm Typologies

Sampling adequacy analysis yielded a KMO statistic value of 0.661 indicating that the data was suitable for EFA (Kaiser, 1970). After eliminating variables exhibiting low variation, 11 variables were entered into EFA. Factor analysis resulted in five factors, accounting for 66% of the total variability being retained (Table 2), while cluster analysis yielded a 4-cluster solution (Table 3). Table 3 indicates that from the *p*-value of the *F*-test, the clusters differed significantly with respect to the weights assigned for the extracted factors.

**Table 2:** Varimax-rotated factor matrix of determinants of smallholder dairy farming systems.

	Extracted factors				
	1	2	3	4	5
Total off-farm income	0.036	0.085	-0.037	0.358	0.086
Total land area owned	0.066	0.027	0.012	**0**.**344**	-0.075
Milk productivity per cow/year	0.455	**0**.**552**	-0.066	0.089	-0.031
Proportion of milk output sold	0.002	**0**.**513**	0.038	0.077	0.084
Number of deworming exercise per year	-0.125	-0.014	0.280	0.174	0.206
Number of tick control exercises per year	0.350	0.074	0.070	0.075	0.053
Proportion of months in a year when Napier grass was purchased	0.066	0.040	0.016	-0.011	**0**.**284**
Proportion of months in a year when oil seed by-product was used	**0**.**656**	0.121	-0.160	0.009	0.073
Proportion of months in a year when bran was used	**0**.**633**	0.197	-**0**.**435**	0.131	0.041
Proportion of months in a year when maize germ was used	-0.014	0.031	**0**.**423**	-0.050	-0.001
Number of lactating cows owned	0.300	**0**.**513**	-0.004	0.070	0.025

*Extraction method: principal axis factoring. Rotation method: varimax with Kaiser normalization. Bolded values are the highest for each extracted factor and represent the determinants with the highest loading*.

**Table 3:** Factor loadings for various production system variables used to define management clusters.

	*F*-test
	*P*-value	Cluster 1	Cluster 2	Cluster 3	Cluster 4
		(*n* = 175)	(*n* = 214)	(*n* = 156)	(*n* = 109)
		27%^∗^	33%	24%	16%
Supplementation intensity and diversity	<0.0001	0.267	0.651	-**0**.**756**	-**0**.**624**
Milk productivity and sale	<0.0001	-**0**.**382**	**0**.**612**	-**0**.**701**	**0**.**414**
Maize germ	<0.0001	-**0**.**364**	-0.252	0.267	**0**.**697**
Wealth	<0.0001	-0.045	0.205	-0.170	-0.086
Purchase of Napier grass	<0.0001	-0.066	0.054	-0.055	0.078
*Further profiling*					
Number of cattle owned	<0.0001	3.114	4.061	2.679	3.376
Sale of milk to non-chilling cooperative	<0.0001	0.011	0.037	0.000	0.000
Sale of milk to chilling cooperative	<0.0001	0.017	0.080	0.000	0.165
Sale of milk to individual customers	<0.0001	0.080	0.410	0.013	0.414
Sale of milk to private traders	<0.0001	0.011	0.208	0.000	0.200
Total milk quantity produced	<0.0001	5.930	14.425	2.298	8.110
Rungwe district	0.395	0.857	0.869	0.141	0.183
Lushoto district	0.395	0.143	0.131	0.859	0.817

*P-values compare the difference between clusters with regards to weights of the factors. ^∗^Percentage of household. Bolded values are the highest for each extracted factor and represent the determinants with the highest loading*.

Cluster one contained about 27% of households consisting of “medium-feed-low-output subsistence oriented dairy farmers,” characterized by low productivity and sale of milk as well as low use of maize germ supplement. Majority of households were grouped in cluster two, which had 33% of households that were “feed intensive commercially oriented dairy farmers.” Households in this cluster used a diversity of supplements such as maize bran and oil seed by-products. These households were also characterized by higher milk sales. Cluster three, which accounted for about 24% of the sampled households consisted of “low-feed low-output subsistence oriented dairy farmers” being characterized by low diversity and intensity of supplement use. Cluster four accounted for 17% of the households which exhibited higher intensity in the use of maize germ but less diversity and intensity of usage for other supplements. These “Maize germ intensive semi-commercial dairy farmers” also had moderate milk productivity and sale.

Households from Rungwe district in the Southern highlands were grouped in clusters one and two, while households from Lushoto district in the Northern highlands were grouped in clusters three and four. The more intensive and commercially oriented farmers in Rungwe also recorded higher overall milk production as did the more intensive and semi-commercial dairy farmers in Lushoto. The disparate classification of households for the two sites in distinct clusters was largely related to the feeding plane and commercial orientation differences between these two sites.

### Genetic Diversity and Admixture

#### Minor Allele Frequencies (MAF)

**Figure 1:**
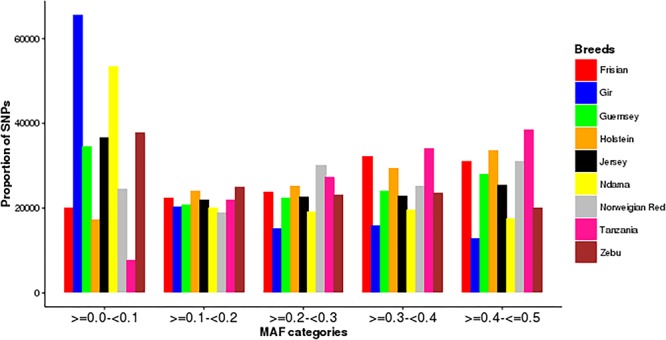
Distribution of minor allele frequencies (MAF) in the study populations.

The distribution of minor allele frequencies in each breed is presented in Figure 1. The Tanzanian population had the highest proportion of the SNPs with high MAF (>0.3). In contrast, the Gir and N’Dama had the highest proportion of SNPs in the lowest MAF band.

#### Admixture Analysis

**Figure 2:**
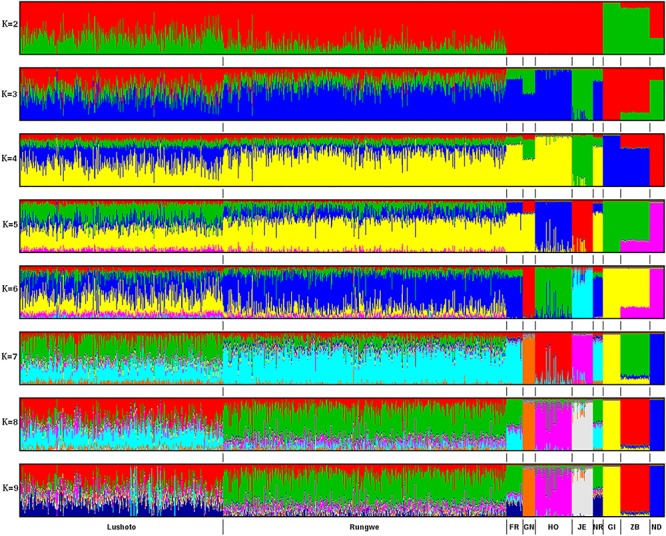
ADMIXTURE bar plots of breed proportions (*K* = 2 to *K* = 9), with *K* representing the optimal number of discrete breeds. Each animal is represented by a vertical line divided into *K* colored segments representing the estimated fraction belonging to each cluster. Short vertical lines at the bottom of each horizontal bar delimit individuals of different populations. Tanzania cattle populations are divided according to the sampling locations, Lushoto and Rungwe, respectively. Reference breeds are labeled as Friesian (FR), Guernsey (GN), Holstein (HO), Norwegian Red (NR), Jersey (JE), Gir (GI), Zebu (ZB), and N’Dama (ND).

Results from ADMIXTURE runs for *K* = 2 to *K* = 9 are presented in Figure 2. Seven clusters were deemed the most optimal given that increasing *K* to 8 did not reveal any new distinct breed clusters or patterns. Based on available genotypes, Friesian and Norwegian Red breeds could not be distinguished apart and formed one cluster. The breed composition of the Tanzanian cattle was largely influenced by Friesian and Norwegian Red breeds. Overall, the predicted exotic taurine breed content (dairyness) in the Tanzania population varied from 7 to 100% and averaged 70%. The subpopulation of cows from Rungwe showed higher levels of taurine admixture (mean 78.3 ± 13%; *n* = 489) than the Lushoto subpopulation (mean 56.4 ± 16%; *n* = 346).

#### Breed Group and Breed Type Definition

Based on the admixture results, the proportion of genes for Holsteins (HOL), Norwegian Red Friesians (RED), Jersey (JER), and Guernsey (GUE), Zebu, N’Dama, and Gir were determined for each of the 539 cows with daily milk records. Initially the percentage dairyness (which is a sum of gene proportions derived from the international dairy breeds used as references) in each animal was computed as the proportion of genes for HOL, RED, JER, and GUE in an animal as determined by the admixture analysis. This was based on the assumption that these four breeds are primarily dairy animals compared to the Zebu, N’Dama, and Gir. Four classes of cows were then created on the basis of the percentage dairyness: animals with >84%, 84–75%, 74–35%, and <34% dairyness, which roughly corresponds to pedigree animal, F2 cross, F1 cross and a backcross or indicine animal, respectively. Within each of the four classes, animals were then grouped on the basis of the order of the breed or breeds with accounted for 76% of the genes in each animal (Table 4). For instance, considering animals with >84% dairyness in Table 4, animals classified as group 1 (RED–GUE) implies the genes from RED or genes from the RED and then GUE or genes from the RED and then Jersey accounted for more than 76% of genes in the animal with the highest proportion coming from the RED. Whereas for animals classified as group 4 (Zebu–RED), genes for the Zebu or genes from the Zebu and then Gir or genes from the Zebu and then the RED or genes from the Zebu and then HOL breed accounted for more than 76% of genes in the animal but with the Zebu accounting for the highest proportion of genes. Note that the choice of 76% genes as the proportion contributed by one or more breeds in classifying animals to breed types was arrived after trying several values so as to get an optimal distribution of genotypes. On the basis of the results in Table 4, nine breed types were defined based on the percentage of the dairyness and the order of breeds accounting for most of the genes in the animal.

**Table 4:** Number of cows included in the analysis, grouped based on a combination of breed composition and percent dairyness.

	Dairyness class (%)			
Breed group	>84%	84–75%	74–35%	34–0%
1. RED–GUE: [Norwegian Red–Friesian (RED), RED–Guernsey, RED–Jersey]	35	7	–	–
2. RED–HOL (Holstein–RED, RED–Holstein)	65	33		–
3. RED–Zebu (RED–Zebu, RED–N’Dama)	13	94	148	–
4. Zebu–RED (Zebu, Zebu–Gir, Zebu–RED, Zebu–Holstein)	–	–	136	21
Total	113	134	284	21

#### Performance and Breed Suitability Assessment

The determination of performance for each breed type and their suitability in the four management clusters was based on the mean values for milk yield computed using the solutions of management clusters nested within the breed types from the fixed regression model as well as the mean breeding values and solutions of permanent environmental effects of each cow in the management system. The distribution of cows based on their dairyness and breed composition is shown in Table 4. Given the average dairyness of 70%, majority of the animals had a breed composition in the 74–35% dairyness range. Most animals were predominantly crosses between Friesian-Norwegian Red breeds and local Zebu cattle.

Generally, the milk yield obtained from the study cows was low averaging 5.90 l per day. The mean daily milk yield for cows in Lushoto was 4.69 l while that of Rungwe was 6.61 l. Cows in breed group 4 (Zebu–RED crosses) had the lowest milk yields ranging between 1.4 and 3.5 l per day (Table 5). Given that this group consisted of cows with the highest proportion of Zebu genes and that the East African Zebu is not improved for milk yield, the low milk yield conforms to expectations. Additionally, majority of low dairyness cows (43% of all Zebu–RED crosses) were kept in the low-feed–low-output management system. Farmers practicing low-feed–low-output subsistence dairy farming were also the only ones who kept animals with dairyness <34% as well as not having animals in the >84% dairyness category. The RED–GUE crosses tended to be the best performing with a narrower range of performance (4.7–6.8). However, these crosses were very few and were not well represented in all management clusters. The RED–HOL group were second highest with a yield range of 3.9–6.7 l per day. The third best group was the RED–Zebu, which had the widest range of performance at 2.1–7.2 l per day. This group also had the highest yields for the *medium-feed–low-output subsistence-oriented* management system. Raw means and means corrected for fixed effects are provided in Table 3.

**Table 5:** The mean daily milk yields (±SD) of various breed groups of varying dairyness in different management clusters.

Breed
group	Management cluster 1				Management cluster 2				Management cluster 3				Management cluster 4			
Mean averages from raw daily milk yield												
	**Dairyness class**												

	**I**	**II**	**III**	**IV**	**I**	**II**	**III**	**IV**	**I**	**II**	**III**	**IV**	**I**	**II**	**III**	**IV**

RG	5.75 ± 3.15	–	–	–	8.35 ± 3.29	–	–	–	–	–	–	–	8.192 ± 2.56	–	–	–
RH	5.75 ± 2.14	7.06 ± 4.21	–	–	7.31 ± 3.36	7.83 ± 2.79	–	–	–	5.040 ± 1.80	–	–	–	7.784 ± 4.42		–
RZ	7.14 ± 3.21	6.00 ± 3.23	5.62 ± 2.66	–	9.29 ± 3.67	6.83 ± 2.97	6.18 ± 3.01	–	–	4.77 ± 2.44	4.03 ± 2.51		–	6.93 ± 2.83	5.96 ± 2.88	–
ZR	–	–	4.03 ± 2.51	–	–	–	5.53 ± 2.60	–	–	–	3.36 ± 1.49	2.797 ± 1.28	–	–	4.38 ± 202	–

**Mean averages for adjusted daily milk yield**												

RG	4.76 ± 1.33	–	–	–	6.88 ± 1.29	–	–	–	–	–	–	–	5.892 ± 1.22	–	–	–
RH	3.92 ± 1.15	4.85 ± 1.33		–	5.70 ± 1.29	6.05 ± 1.25	–	–	–	3.942 ± 1.10			–	6.79 ± 1.36	–	–
RZ	7.28 ± 1.19	4.15 ± 1.27	4.03 ± 1.19	–	7.10 ± 1.30	5.48 ± 1.28	4.44 ± 1.26	–	–	2.19 ± 1.06	2.70 ± 1.25		–	4.97 ± 1.28	4.08 ± 1.22	–
ZR	–	–	2.49 ± 1.19	–	–	–	3.58 ± 1.17	–	–		1.95 ± 1.13	1.48 ± 1.13	–	–	2.78 ± 1.16	–

*I = >84%, II = 84–75%, III = 74–35%, and IV = <35%; management clusters 1, 2, 3, 4 corresponds to “*medium-feed–low-output subsistence oriented dairy farmers*,” “*feed-intensive commercially-oriented dairy farmers*”; “*low-feed–low-output subsistence oriented dairy farmers*” and “*maize-germ-intensive semi-commercial dairy farmers.”* Breed group (the combination of breeds that contribute 76% of the breed makeup). RG, RED–Guernsey; RH, RED–Holstein; RZ, RED–Zebu cross; ZR, Zebu–RED. Means were adjusted based on the fitted G-BLUP model*.

Table 6 indicates the breed composition of the top 10 cows in terms of EBVs in each management group. Each of the four management clusters had a total of 130, 203, 105, and 101 cows, respectively, such that the top 10 cows represented the top 8, 5, 10, and 10%, respectively, in each group. For all management clusters (except the *feed-intensive commercially-oriented* management group), cows whose composition was dominated by crosses of the Friesian-Norwegian Red and Zebu (RED–Zebu, either as ZR or RZ genotypes) dominated the list of top 10 animals based on EBV ranking (Table 6). Conversely, crosses of Friesian-Norwegian Red and Holstein (RHZ, RH, RZH) featured mostly in the *feed-intensive commercially-oriented* and *maize-germ-intensive semi-commercial* management clusters.

**Table 6:** The top 10 cows in breeding values for milk yield in each management group with their percentage dairyness and breed composition.

	Herd management 1			Herd management 2			Herd management 3			Herd management 4		
Rank	Breed	D%	EBV^∗^	Breed	D%	EBV	Breed	D%	EBV	Breed	D%	EBV
1	RZH	76	2.33	RZ	76	2.42	RZG	64	2.31	RZG	85	1.90
2	HRZ	75	2.20	RH	90	2.41	RZH	70	1.61	RZG	70	1.58
3	R	83	2.17	RJ	85	2.38	ZR	43	1.13	RZ	72	1.18
4	R	89	2.05	RH	87	2.31	ZR	40	0.98	RZ	79	1.16
5	RZH	76	1.97	RH	85	2.14	ZR	39	0.83	RZ	65	1.13
6	RZ	76	1.56	RZ	79	2.01	RHZ	78	0.82	RZJ	67	1.04
7	RZ	55	1.55	RHZ	75	2.00	ZR	48	0.80	ZR	46	1.01
8	RZ	55	1.33	RZ	84	1.90	ZHR	65	0.77	RZH	65	0.87
9	RZ	78	1.26	RZ	57	1.88	ZRH	55	0.73	RZ	62	0.87
10	RZ	72	1.22	RH	85	1.86	ZRH	62	0.72	ZRG	55	0.78

^∗^*Liters per day. Breed is the breed group (the combination of breeds that contribute 76% of the breed makeup), with the first letter representing the breed having the highest proportion. D% represents percent dairyness (the cumulative proportion of taurine dairy breed composition in the cow). RZ, RED–Zebu cross; RH, RED–Holstein; RZH, RED–Zebu–Holstein cross; RZJ, RED–Zebu–Jersey; RHZ, RED–Holstein–Zebu; HRZ, Holstein–RED–Zebu; RJ, RED–Jersey; ZR, Zebu–RED; ZRH, Zebu–RED–Holstein; ZHR, Zebu–Holstein–RED; ZRG, Zebu–RED–Guernsey*.

#### Genetic Parameters

Following variance component analysis, the direct additive heritability estimate obtained for milk yield was 0.24 ± 0.13 while repeatability was 0.32 ± 0.04. The heritability estimates fell within the range (0.18–0.51) estimated for taurine cattle (Van Tassell et al., 1999). The genetic parameter estimates were well within values obtained from tropical smallholder systems (Msanga et al., 2000).

## Discussion

The purpose of the project was to characterize the smallholder dairy system and identify how various breed types performed under varying management clusters. By identifying the exact breed composition of target cows and associating the observed profile with individual animal productivity, it is envisaged that appropriate recommendations can be made for farmers and others intending to maximize productivity of these systems.

### Management Group Clustering

Central to matching breed types to production environments is the need to characterize the production environments. This is critical because most smallholder dairy farmers have small herd sizes averaging two to three animals. Additionally, the management practices in these farms are very divergent, making evaluation of performance potentially difficult. A strategy to overcome such heterogeneity in management practices is to find commonalities in practices between households. These clusters would then represent some fairly homogenous groups of households (ostensibly undertake somewhat similar management practices) within which the performance of extant cohorts of animals can be evaluated. Each cluster would then be considered a management group. This was achieved through first a factor analysis of various variables collected in the baseline survey such as farm income, land area owned, and type of feed used, among others followed by a cluster analysis of the five extracted factors. Given the four management clusters defined, many subliminal factors are implied and contribute to the observed differences in the productivity of households therein. The variety and intensity of supplementation characterized in the feed-intensive commercially oriented management system and the maize germ intensive semi-commercial dairy farmers implied more labor input, in the search and preparation of the materials. Additionally, given that most of these materials are mostly not purchased but sourced from own farms, variable sources and types of supplements would reflect a larger land area planted and potentially higher household income obtained from the sale of a diverse crop base.

Classification based on inter-farm differences can potentially enable identification of farms with similar practices and circumstances for which a given recommendation would be broadly appropriate (Byerlee and Collinson, 1980). Similarity among households within a management system is no doubt determined by constraints and opportunities faced by the farmers and these are expected to vary according to agro-ecological and socioeconomic conditions under which farmers operate. Even within the same agro-ecological conditions, individual households may still differ due to socio-economic conditions and inherent knowledge. There will often therefore be need for targeted solutions that take into account diversity in farm resource endowment and farm practices in spite of similarities in agro-ecological conditions. This fact is demonstrated by farmers in the same geographic regions being classified in disparate management clusters. Membership in each of these four management clusters was driven by feeding practices, productivity and commercial orientation of dairy farm households.

### Admixture and Breed Composition

In order to establish the breed composition of the animals, we collected blood and hair samples from a total of 839 cows from Lushoto and Rungwe in Northern and Southern highlands of Tanzania, respectively. The choice of the genotyping platform used (the Geneseek Genomic Profiler Dairy) was informed by the need to minimize the cost of genotyping, as well as access genotypes that can be pooled with available reference genotypes, which were genotyped by the Illumina 700K SNP array. However, the SNP array that was used to genotype the animals had no power to discriminate between Norwegian Red and Friesian breeds. Additionally, the panel had a significant number of polymorphisms that had very low minor allele frequencies in indicine breeds, indicating that it may lack the power to detect subtle difference between genetic signatures derived from the indicus background. This ‘ascertainment’ bias compromises the definitive determination of the exact breed composition, especially relating to indicine genetic composition. However, for our purposes, the goal of determining dairyness was largely achieved.

Breed groups were defined based on a combination of percentage dairyness and the number of breeds making up 76% dairyness. The dairyness classes represent grade levels with respect to crossbreeding with indigenous breeds. Typically, an animal is assigned to a specific breed if its genes are composed of at least 87.5% from that breed. In our case, using this as a cut-off resulted in skewed distribution of animals and genotypes. The best possible distribution was arrived at with a cutoff of 76%. On the basis of this, four breed groups were defined, giving a total of nine breed types when combining dairyness and breed group. It should be noted that based on the genotyping array used, it was not possible to distinguish between the Norwegian Red and the Friesian breeds. The foregoing discussion will treat these two breeds as equivalent.

Based on the results from breed composition analysis, it is evident that the range of admixture in Tanzanian dairy cattle is quite wide given the spectrum of taurine introgression observed. For cows in Lushoto, the proportion of taurine genes ranged from less than 20% to greater than 85%. In Rungwe more than 95% of all cows had a taurine gene composition of above 50%. The variety of breeds used in crossbreeding was quite narrow compared to what has been observed in other East African countries (Weerasinghe, 2014). The predominant breed was the Holstein-Friesian, with a bias toward a Friesian signature. There appeared to be limited or no use of the Jersey, Guernsey or Ayrshire breeds. These breeds are often smaller than the Holstein and would be easier to handle in smallholder farming systems given their lower feed requirements. This result is consistent with the dominant importation of black and white genetics as the main breed for dairy farming. However, it was surprising to see that the predominance of Holstein, as expected is not reflected in the breed composition results. Holstein is the main breed imported into East African dairy systems.

Despite the fact that Lushoto and Rungwe are quite similar with regard to elevation and climate, (both being in highland areas), the fodder density, feed availability, and farmer practices were quite different. Additionally, even though we did not collect body weight or heart girth data on the study cows, differences in animal stature were evident. Cows in Lushoto were smaller, were more horned, and had prominent dewlaps compared to those in Rungwe. Based on the breed composition results observed, and the fact that on average, Lushoto animals had about 50% Zebu signature, the differences can be confidently attributed to differential taurine gene introgression. The feed density available in Lushoto and associated management practices can hardly support higher grade exotics for majority of the farmers, who would prefer lower grade crosses that require less rigorous maintenance. Additionally, the terrain in Lushoto is also quite steep in many places, reducing capacity of the land to hold enough fodder for the animals, while also presenting a soil nutrition challenge. Soils in Lushoto are less fertile compared to Rungwe and hence the feed mix available would be poorer. In Lushoto, most farmers feed crop residues (maize stover, guatemala grass, and grain products), which are offered seasonally, mostly after harvest. However, farmers in Rungwe have a larger diversity including purchased feeds, banana stalks, Napier grass among others as the main feed source.

### Recommendations for Appropriate Breed Type

Usually, milk yields in small holder farms do not follow the typical lactation curve mostly due to poor management associated with erratic sub-optimal feeding and other constraints found in tropical production systems. To deal with this problem, and to increase the flexibility of resultant curves, a single trait animal model with Legendre polynomials of order 3 (with fixed curves nested within breed types) was fitted (Supplementary Figure S1). Legendre polynomials have been shown to perform well in such situations (Eva Strucken, personal communication). The mean production seen in Tanzania (5.9 l per day) is very similar to what has been recorded in Kenya and Uganda. A similar study carried out over a 2-year period in Kenya and Uganda (and with 39,000 milk yield records) resulted in very similar performance in smallholder systems, averaging 5.39 and 5.62 l, respectively (Unpublished). Smallholder farmers are the backbone of the dairy sector in Tanzania and East Africa. It is generally agreed that a successful dairy operation should utilize improved breed types given the low productivity of local zebu cattle. This desire for increased production drives farmers into crossbreeding, the general sense being that a pure bred exotic animal isn’t suitable either for a majority of smallholder farmers. However, there is little information or evidence to support what should be the ideal breed type for various smallholder production situations. By evaluating the performance of various breed types within diverse management clusters, it is possible to provide general recommendations of the breed type most effective for each circumstance.

Given the estimated breeding values obtained in this study and the top 10 ranked animals, it is clear that Holstein genetics are not well suited for the smallholder system of the kind profiled in this study. It is difficult to say whether the alternative is Friesian or Norwegian Red given the ineffectual separation of these two breeds in the study. However, we expect that since there is significant representation of Friesian in the Norwegian Red breed, hence the lack of differentiation with the number of markers on the GGP SNP array. However, based on the breed utilization pattern in the region, the breed in question would mostly be Friesian, since most farmers either prefer or have easy access to the black and white cattle. A similar phenomenon was observed by Weerasinghe (2014), where exclusion of Ayrshire as a reference breed resulted in Ayrshire animals having higher Jersey or Guernsey composition. However, that animals with substantial Holstein background were performing inferior to smaller bodied alternatives is not surprising. Anecdotal evidence and common sense would dictate that in the face of limiting feed resources, sub-optimal management practices and extant disease pressure in smallholder systems, cows that are smaller framed would be preferred, not least because of the lower feeding requirements. However, as farmers chase larger milk yields, preference has fast shifted to Holsteins and their promise of huge milk production. One of the most illuminating outcomes in this analysis was the fact that some of the Zebu–RED cows, those of the low dairyness class, were ranked amongst the best performers in some management clusters. These animals, with dairyness less than 60%, typify the benefits that may be derived through regular performance recording and evaluation. It would be interesting to identify the genetic signature of such animals, because they would best exemplify the model cow for smallholder systems – resistant to diseases, hardy enough to withstand poor feeds and ravages of the tropical smallholder system, but still competitive in terms of milk yield. However, because farmers do not routinely collect performance records, nor is there a consistent mechanism for performance evaluation, any hidden gem in the national herd is soon lost in pursuit of higher yields through inappropriate upgrading.

The results obtained in this study seem to suggest that the RED–Zebu with exotic genes between 75 and 85% are the most appropriate genotype for these systems followed by the RED–GUE. For farmers in the feed-intensive-commercially oriented dairy management group, the RED–HOL or RED–GUE crosses with at least 75% exotic genes were the best performing cows. Farmers in the low-feed–low-output subsistence oriented dairy farming would be best served with animals with breed composition of no more than 65% RED genes. This means that dairy farmers who are able to provide the feeding plane and management inputs for the Holstein, can still be well served by that breed type. However, this group does not represent the vast majority of smallholder farmers.

Collecting data from smallholder dairy systems is an enormously expensive and tasking exercise. Typically, routine collection of test day milk yield records does not happen and such data is the preserve of research institutions. There is no incentive for collecting such data for smallholder farmers because genetic evaluation programs are lacking. Where these systems exists, they are only done for large scale commercial farmers with large herd sizes. The extremely small number of animals kept by smallholder farmers (most farmers keep two dairy cows), the cohort sizes are too small for meaningful genetic evaluation to be undertaken. Additionally, smallholder farmers do not raise their own animals for replacement, being content to buy replacement stock from established farms when needed. These limitations contributed greatly to the low data volumes experienced in this study. With the limited data available, we were able to demonstrate that combining genomic data with lactation and other production records can be a powerful way of identifying appropriate genotypes for farmers given their extant management system. The results obtained in this study can serve as a basis to inform the development of the dairy sector in Tanzania. This is particularly important because the Tanzanian government has resolved to increase the number of improved dairy cattle to three million head and milk production from 1.6 billion to 6 billion liters annually in the next 10 years. Knowledge of what breed combinations are best suited for which production systems is critical and will determine the success of this ambitious goal.

The recommendations of breed types most suitable for the management clusters described in this study reflects only the sample set which was surveyed and highly related systems and cannot be generalized across the diversity of smallholder farming enterprises. These are variable and are immensely influenced by socio-economic parameters, market orientation, available feed resources as well as other agro-ecological factors. Additionally, data for this study was collected over a 7-month period, and not a full lactation for each animal. The study duration was short and sample size limited. These results would gain tremendously from increasing the number of lactations, the number of test day records as well as larger sample sizes to solidify the recommendations proffered herein. However, such a study would be very costly. In practice, milk yield recording is not an entrenched practice in smallholder dairy systems. Such data collection would primarily be driven by hired enumerators, making the cost very high. Owing to limited funding and competing needs for available resources, data can only be collected for limited durations of time.

The recommendations made in this study are based solely on performance in terms of daily milk yield and do not account for other important issues such as cost of health treatment, reproductive management or feed provision. An economic analysis that accounts for all these additional variables will be useful in defining the most profitable genotype for each system.

## Conclusion

The use of SNP data and genomic relationships for the animals under study enabled performance evaluation of milk yield data in smallholder dairy farms without the need for pedigree records. The breeding values estimates so obtained were instrumental in determining that the RED–Zebu breed type with exotic genes between 75 and 85% was the most appropriate genotype for majority of the management clusters except the high input clusters. Given that majority of smallholder farmers operate in circumstances where the intensity of input (especially feed) provision is quite limited, the recommended breed type would be the most applicable on a wide scale. These results indicate that matching breed type to production management group is central to sustainable intensification and maximizing productivity. The observations made in this study will serve as a basis to inform the development of the dairy sector in Tanzania and Eastern Africa at large.

## Ethics Statement

This study was performed following the International Livestock Research Institute (ILRI) Institutional Animal Care and Use Committee (IACUC) guidelines, with approval reference number 2014.35. Animals were handled by experienced animal health professionals to minimize discomfort and injury.

## Author Contributions

FM conceived the project, designed the study, and obtained funding. AK, DN, and MA were involved in collection of field data. JR, EC, FM, and RM analyzed the data and contributed to drafting individual segments for the analyses. FM consolidated inputs and drafted the manuscript. JR, YZ, MA, and RM made suggestions and corrections. All authors read and approved the final manuscript.

## Conflict of Interest Statement

FM and EC were employed by USOMI Limited, a private company during manuscript preparation. AK was employed by Badili Innovations during the manuscript preparation. All the work relating to this research was done prior to their employment by the respective companies. All other authors declare no competing interests.
