# Supplementary material for: Performance Evaluation of Highly Admixed Tanzanian Smallholder Dairy Cattle Using SNP Derived Kinship Matrix
Source: Front Genet. 2019 Apr 26;10:375. doi: 10.3389/fgene.2019.00375 (PMC6498096; doi:10.3389/fgene.2019.00375)
Supplement: Supplementary file 1 [file Table_1.docx]

Fitted plots of fixed lactations using Legendre polynomials of order 3 for cows of breed group RED-GUE of >84% exotic genes (A), RED-HOL with 84-75% exotic genes (B), RED-Zebu with 74-35% exotic genes (C) and Zebu-RED with less than 35% exotic genes (D).
