# Supplementary figures and images for: Performance Evaluation of Highly Admixed Tanzanian Smallholder Dairy Cattle Using SNP Derived Kinship Matrix
Source: Front Genet. 2019 Apr 26;10:375. doi: 10.3389/fgene.2019.00375 (PMC6498096; doi:10.3389/fgene.2019.00375)

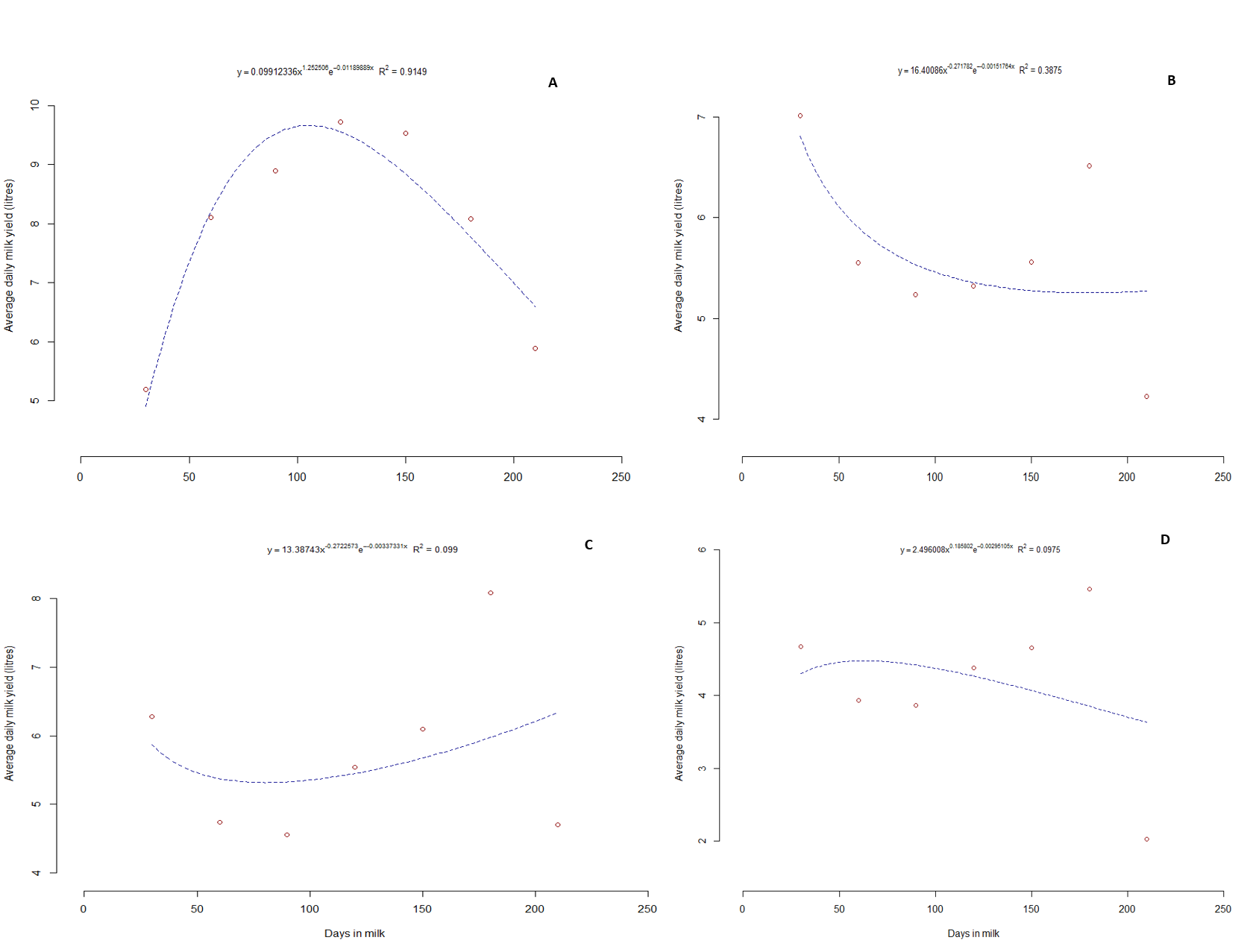

Supplement: Supplementary file 2 [file Image_1.tif]
